# Dorso-ventral skin characterization of the farmed fish gilthead seabream (*Sparus aurata*)

**DOI:** 10.1371/journal.pone.0180438

**Published:** 2017-06-30

**Authors:** Héctor Cordero, Diana Ceballos-Francisco, Alberto Cuesta, María Ángeles Esteban

**Affiliations:** Fish Innate Immune System Group, Department of Cell Biology and Histology, Faculty of Biology, University of Murcia, Murcia, Spain; Universitat Politècnica de València, SPAIN

## Abstract

The skin is the first barrier of defence in fish, protecting against any external stressor and preserving the integrity and homeostasis of the fish body. The aim of this study was to characterise gilthead seabream skin by isolating cells and studying the cell cycle by flow cytometry, to study the skin histology by scanning electron microscopy and the transcription level of some immune-relevant genes by RT-PCR. Furthermore, the results obtained from samples taken from the dorsal and the ventral part of the specimens are compared. No differences were observed in the cell cycle of cells isolated from the dorsal and ventral zones of the skin or in the gene expression of the genes studied in both epidermal zones. However, the epidermis thickness of the ventral skin was higher than that of the dorsal skin, as demonstrated by image analysis using light microscopy. Besides, scanning electron microscopy pointed to a greater cell size and area of microridges in the apical part of the dorsal epidermal cells compared with ventral skin epidermal cells. This study represents a step forward in our knowledge of the skin structure of an important farmed teleost, gilthead seabream, one of the most commonly farmed fish worldwide. Furthermore, for functional characterization, experimental wounds were carried out comparing the wound healing rate between the dorsal and ventral regions of skin over the time. The results showed higher ratio of wound healing in the ventral region, whose wounds were closed after 15 days, compared to dorsal region of skin. Taking into account all together, this study represents a step forward in our knowledge of the skin structure and skin regeneration of an important farmed teleost, gilthead seabream, one of the most commonly farmed fish worldwide.

## Introduction

Gilthead seabream (*Sparus aurata* L.) is one of the most widely farmed fish species, world production increasing from 77,510 tonnes in 2002 to 158,390 tonnes in 2014 (FAO, 2016). However, due to intensive fish farming, the most widely used method of fish production [1], fish are frequently exposed to external aggressions. Skin, as the outermost organ of the body, is important in this respect, since it not only acts as a physical, mechanical, biological and chemical barrier in teleosts but also confers biological and immunological protection by preventing the entry of pathogens into the body [2].

Teleost skin, in sharp contrast to the skin of mammals, is a non-keratinised tegument, with living cells. The outermost layer of cells forms the epidermis, which is of ectodermal origin and responsible for secreting the skin mucus through goblet cells [3]. The surface of the most external epithelial cells has special structures called microridges, whose functions are poorly understood [4]. The epidermis is separated from the next inner layer (dermis) by an acellular basement membrane. The dermis, which is derived from the mesoderm, is thicker, vascularised, and can be divided into two clear sublayers: (i) the *stratum spongiosum* with vascular and neural components where scales are embedded and most of the chromatophores are located; and (ii) the *stratum compactum* which mostly consists of a dense matrix of collagen fibres [5]. Lastly, an internal layer, the hypodermis, is mainly (but not exclusively) a fat reservoir r composed of adipose cells that link the skin with the underlying muscle.

For adaptation to the aquatic environment, fish skin is covered by mucus that serves to maintain homeostasis and integrity [6]. In recent years, most fish skin research has focused on the mucus layer and gilthead seabream mucus is no exception in this respect. The physico-chemical parameters of skin mucus, such as viscosity, density, conductivity, pH or redox potential, have been described [7]. The presence and abundance of enzymes and/or immunologically active molecules, including proteases, antiproteases, esterase, phosphatase alkaline, peroxidase, lysozyme, immunoglobulin M (IgM) or bacteriostatic peptides, have also been demonstrated in gilthead seabream skin mucus [8,9]. Furthermore, the proteome map of skin mucus in several teleosts [10,11], including gilthead seabream [12,13], has been characterized, as well as the proteomic profiling after dietary supplementation and/or stress [14]. However, few classical microscopic studies have been made of fish skin cells [15,16].

Few studies have taken into consideration possible differences between the epidermis from different parts of the fish body. One such paper evaluated the role of agouti-signalling protein (ASC) in the different dorsal-ventral pattern of skin pigmentation of fish [17]. At transcription level, only one study from isolated skin cells of Atlantic cod revealed changes in some immune-related (*lyz*, *il1b*) and stress-related (*sod*, *cat*) genes after probiotic-pathogen interactions [18]. In gilthead seabream skin explants, we recently reported significant changes in the cytokine transcript profile in the ventral skin (but not in dorsal skin) after probiotic-pathogen interaction [19]. Taking into account all these considerations, the aim of this work was the characterization of the dorsal and ventral skin of gilthead seabream through the isolation of skin cells, histological analysis of skin by light and electron microscopy (including image analysis for measuring the thickness of the epidermal layer, cell size and the area occupied by microridges), as well as to study important immune-markers previously found in skin mucus at transcript level in both regions of the skin. Finally, as a functional approximation, the wound healing ratio was determined over the time in the dorsal and ventral regions of the skin.

## Materials and methods

### Animals

Specimens of the hermaphroditic protandrous teleost gilthead seabream (*S*. *aurata* L.) (80–120 g and 15–22 cm) obtained from a local farm in Murcia (Spain) were kept in re-circulating seawater aquaria (250 L) with a flow rate of 900 L h^-1^ in the Marine Fish Facility at the University of Murcia and allowed to acclimatize for 2 weeks. The temperature and salinity were 22 ± 2°C and 28 ‰, respectively. The photoperiod was of 12 h light: 12 h dark. A commercial diet (Skretting, Spain) was administered at a rate of 2% body weight day^-1^. Fish were anesthetized with 100 mg l^-1^ MS222 prior to sampling the skin in each trial. Mucus was gently removed by a cell scraper before sampling the skin as detailed in the following sections.

The protocol was approved by the Committee on the Ethics of Animal Experiments of the University of Murcia (Permit Number: A13150104).

### Isolation of cells

Samples from dorsal and ventral skin around the central part of the body (n = 4 fish specimens) were cut into small pieces (~ 0.5 cm^2^) after removing the muscle and washing with ice-cold phosphate buffered saline (PBS) supplemented with antibiotics (100 I.U. ml^-1^ penicillin and 100 μg ml^-1^ streptomycin). Then, skin fragments from each fish specimen were placed in keratinocytes medium (Sigma-Aldrich) supplemented with 5 mg ml^-1^ of dispase (Gibco), 5% foetal bovine serum (FBS; Gibco), 10 ng ml^-1^ epidermal growth factor (EGF; Life Technologies), and the aforementioned antibiotics. Skin fragments were incubated on ice with shaking for 3 h to disaggregate the cells. Disaggregated cells were then passed through a 100-μm cell strainer. The previous steps were repeated once with the remaining aggregated fragments. Finally, all the cell suspensions obtained from each skin sample were combined and centrifuged (400 g, 10 min) before resuspending the cells in the same culture medium without dispase. The number of cells was determined in Neubauer’s chambers and cell viability was estimated by the trypan blue exclusion test.

### Light microscopy

The isolated dorsal and ventral skin cells were adjusted to 10^5^ cell ml^-1^ and cytocentrifuged (800 g, 5 min, 4°C). Next, the cells were fixed in methanol for 10 min and stained with Giemsa (Merck) at 10% (v/v) for 60 min, washed with tap water and mounted with DPX (Merck). The slides were then examined under a light microscope (Leica DM6000B) and images were obtained with a digital camera (Leica DFC280) and processed by Leica Application Suite V 2.5.0. Software.

### Cell cycle analysis

Freshly isolated skin cells were resuspended in 200 μl of PBS and 1 ml of a 70% ethanol solution was added dropwise while stirring. After 30 min of incubation, cells were washed and resuspended in 800 μl of PBS. Finally, 100 μl of RNAse (1 mg ml^-1^; Thermo Fisher Scientific) and 100 μl of propidium iodide (PI, 400 μg ml^-1^; Sigma-Aldrich) were added and the mixture was incubated at 25°C for 30 min in the dark. Dorsal and ventral skin cells were acquired by a FACScalibur flow cytometer (Becton Dickinson) and cell cycle analysis was performed for 10,000 events using the ModFit LT™ software (Verity Software House). The experiment was performed twice with duplicate samples.

### Skin histology by light microscopy

Dorsal and ventral skin samples (n = 6 fish specimens) were dissected into pieces (~ 0.5 cm^2^), washed in PBS and fixed in 4% paraformaldehyde for 24 h. Next, samples were decalcified in 0.5 M ethylenediaminetetraacetic acid (EDTA; Sigma-Aldrich) solution for 24 h, dehydrated in increased concentrations of ethanol (Merck) (70% for 24 h, 80% for 30 min, 96% for 1 h, and 100% for 1 h three times), then washed for 1 h three times in isoamyl acetate, and finally embedded in Histoplast Paraffin (Thermo Fisher Scientific). Skin samples were sectioned at 5 μm and stained with hematoxylin-eosin (H&E; Merck), periodic acid–Schiff (PAS; Merck) or trichrome of Mallory (Merck) according to the manufacturer’s instructions. Samples were studied under a light microscope and images were taken. The quantitative analysis of epidermal thickness was performed with Leica QWin software (Leica Microsystems Ltd.).

### Electron microscopy

For scanning electron microscopy (SEM), skin samples from dorsal and ventral zones (n = 6 fish specimens) were washed, fixed according to McDowell and Trump [20] for 7 h, placed in washing buffer containing 0.2 M cacodylate buffer with 8% saccharose (Merck) and post-fixed later in 1% OsO_4_ (Merck) for 1 h and 30 min. Afterwards, samples were dehydrated in acetone (from 30% to 100%, 20 min each), critical-point dried, sputter coated with gold and examined with a Jeol JSM-6100 scanning electron microscope. Images were acquired with the software INCA Suite V4.09 (Oxford Instruments). The quantitative analysis of the cell area and microridge area was performed with Leica QWin software.

### Gene expression analysis

For total RNA isolation, dorsal and ventral pieces of skin (n = 4 fish specimens) were sampled, added to TRIzol^®^ reagent (Thermo Fisher Scientific) and processed as indicated by the manufacturer. The RNA present in samples was then quantified and the purity assessed by spectrophotometry; the 260:280 ratios were 1.8–2.0. The RNA was then treated with DNase I (Promega) to remove genomic DNA contamination. To check the RNA quality, an agarose gel was run with all the samples. Complementary DNA (cDNA) was synthesized from 1 μg of total RNA using the SuperScript IV reverse transcriptase (Life Technologies) with an oligo-dT18 primer, according to the manufacturer’s instructions.

The transcription analysis was carried out by real-time PCR (qPCR). The expression of selected genes was analysed with the 2^−ΔCt^ method [21], which was performed as described elsewhere [10]. Primers are shown in Table 1. Each sample (n = 4) was performed in triplicated in the qPCR study. The specificity of the reactions was analysed using samples without cDNA as negative controls. All qPCR reactions were carried out in duplicate and quantification cycle (Ct) values of target genes were converted into relative quantities using reference genes. So, for each mRNA, gene expression was corrected by both the elongation factor 1 alpha (*ef1a*) and ribosomal protein S18 (*rps18*) RNA content in each sample.

**Table 1:** Oligonucleotides used for qPCR study. Gene symbols follow the zebrafish nomenclature (http://zfin.org/). Each primer pair was designed from each sequence with the corresponding ID or accession number according to the NCBI database (http://www.ncbi.nlm.nih.gov/).

Gene name	Symbol	ID number	Sequence (5´→3´)
elongation factor 1-alpha	*ef1a*	AF184170	F: TGTCATCAAGGCTGTTGAGC R: GCACACTTCTTGTTGCTGGA
ribosomal protein s18	*rps18*	AM490061	F: CGAAAGCATTTGCCAAGAAT R: AGTTGGCACCGTTTATGGTC
immunoglobin T heavy chain	*ight*	FM145138	F: TGGCAAATTGATGGACAAAA R: CCATCTCCCTTGTGGACAGT
immunoglobulin M heavy chain	*ighm*	JQ811851	F: CAACATGCCCAATTGATGAG R: GGCACGACACTCTAGCTTCC
lysozyme	*lyz*	AM749959	F: CCAGGGCTGGAAATCAACTA R: CCAACATCAACACCTGCAAC
caspase 1	*casp1*	AM490060	F: ACGAGGTGGTGAAACACACA R: GTCCGTCTCTTCGAGTTTGC
leucocyte elastase inhibitor	*lei*	FM146914	F: GGTGTGTTGGACAGCATGAC R: CATCACGTGTGACGTCTTCC
heat shock protein 70 kDa	*hsp70*	EU805481	F: AATGTTCTGCGCATCATCAA R: GCCTCCACCAAGATCAAAGA
natural killer enhancing factor 1	*nkef1*	GQ252679	F: CTCCAAGCAATAATAAGCCCA R: TCACTCTACAGACAACAGAAC
natural killer enhancing factor 2	*nkef2*	GQ252680	F: CAAGCAGTAAATGTGAAGGTC R: GATTGGACGCCATGAGATAC
glutathione S-transferase	*gst*	AY362762	F: AAATGGCGGACGTCATCTAC R: CCCACATCTTGATCTCAGCA

### Experimental wounds

One circular wound of 4 mm was performed with a biopsy punch (Stiefel) on the dorsal or on the ventral regions of each fish (n = 3 for each of the regions). To follow the wound healing process, macroscopic images were taken with Olympus FE-4000 camera at day 0, 7 and 15. To measure the area after experimental wounds, pictures were analyzed with Leica QWin image analysis software (Leica Microsystems Ltd.), and expressed in mm^2^. Images of the dorsal and ventral experimental wounds are provided as S1 Fig.

### Statistical analysis

Data were statistically analysed by Student t-test to determine differences between the dorsal and the ventral skin areas using Statistical Package for Social Science (SPSS for Windows; v19) and differences were considered statistically significant when p < 0.05.

## Results and discussion

### Skin cells isolated from dorsal and ventral parts of fish had similar cell cycles

**Fig 1:**
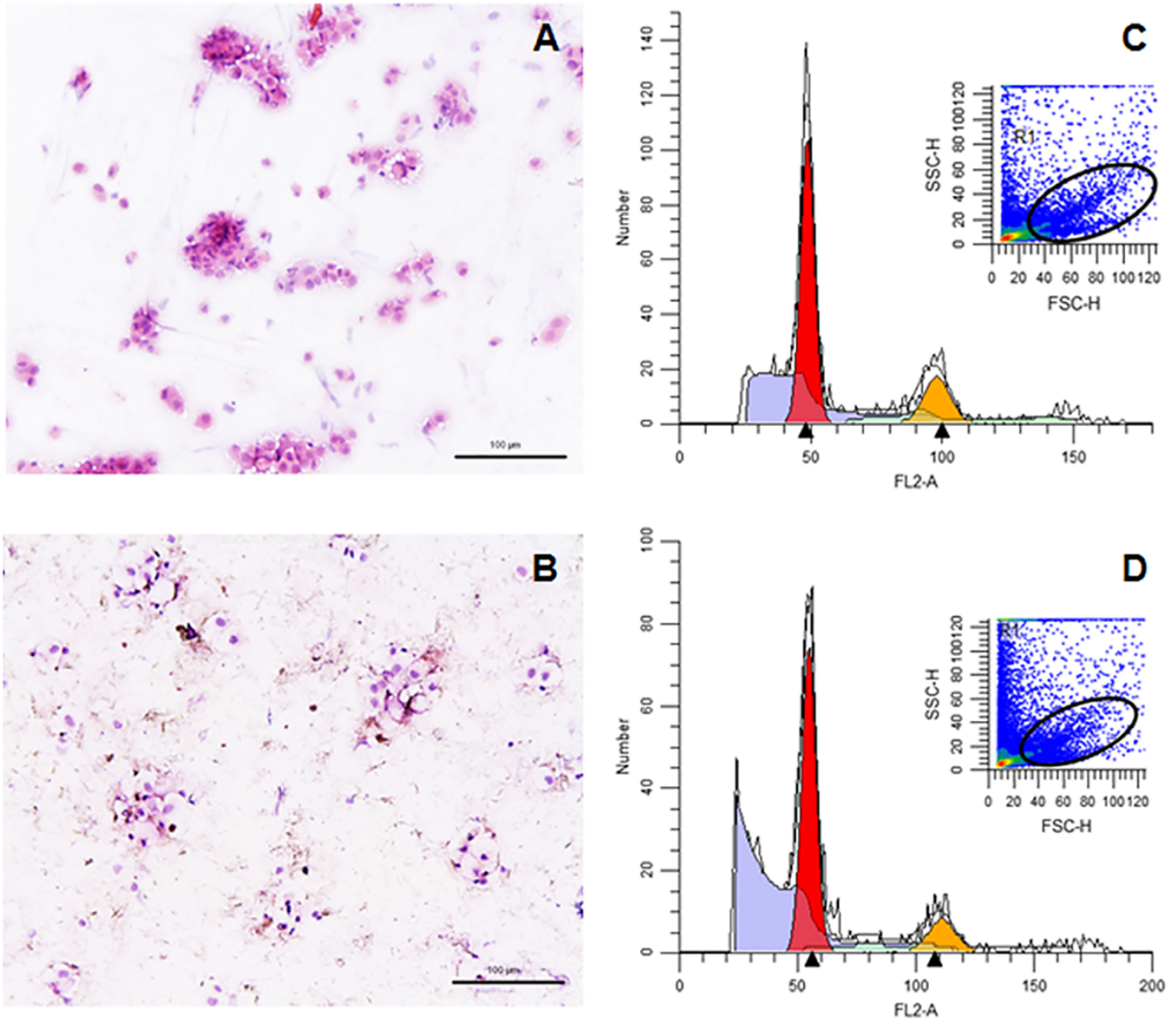
Isolated cells and cell cycle. Representative light microscopy images of freshly isolated skin cells from dorsal (A) and ventral (B) regions of gilthead seabream stained with haemotoxylin-eosin after cytocentrifugation. Cell cycle analysis of isolated skin cells of gilthead seabream from dorsal (C) and ventral (D) zones by flow cytometry. Cells in G0/G1 phases (red), cells in G2/M phases (yellow) and apoptotic cells (blue).

Skin is probably the toughest organ of bony fish since it is responsible for maintaining fish integrity (among other functions [2]). Protocols for isolating fish skin cells are scarce but it is known that they are very difficult to isolate, and only one study has attempted to do so with any success [18]. The study in question showed that in the case of Atlantic salmon skin cells disaggregation, trypsin was less efficient in terms of cell numbers and viability than dispase, the enzyme used in the present research. In our study, skin cells from the dorsal and ventral regions were successfully isolated in gilthead seabream (Fig 1A and 1B) and some differences in the staining pattern were observed between the cells from both regions. Most cells were roughly round with a small and eccentric nucleus. Interestingly, the staining pattern pointed to great differences—eosinophilic or light eosinophilic cytoplasm in the epithelial cells of the dorsal skin and very clear to non-stained cytoplasm in the cells from the ventral area. Strikingly, seabream skin cells were 10–15 μm in size whilst isolated Atlantic salmon skin cells measured 200–300 μm [18] but shared the round morphology and eccentric nucleus. In addition, since epidermal cells of fish skin are actively replicating [22] we evaluated the cell cycle (Fig 1C and 1D). In the dorsal skin, the results showed 76.9% of cells in the G1/G0 phase and 23.1% in the G2/M phase, whilst in the ventral skin the percentages were 83.8% and 16.2% in G1/G0 and G2/M phases, respectively, with no significant differences in the cell cycle between both skin areas. Moreover, apoptosis was more pronounced in ventral cells than in dorsal cells, suggesting that they are more susceptible to the isolation procedure and perhaps to injury. To the best of our knowledge, this is the first time that the cell cycle of skin cells has been compared between different corporal regions. However, widening the study to the cell cycle of skin cells opens up new possibilities for analysing cell regulation in common fish skin infections such as lymphocystis [23] or vibriosis [24], a field with potential applications in fish aquaculture. In this sense, the apoptosis of isolated salmon skin cells increased upon *in vitro* exposure to *Vibrio anguillarum* [18].

### Light microscopy identified different thicknesses in dorsal and ventral skin epidermis

**Fig 2:**
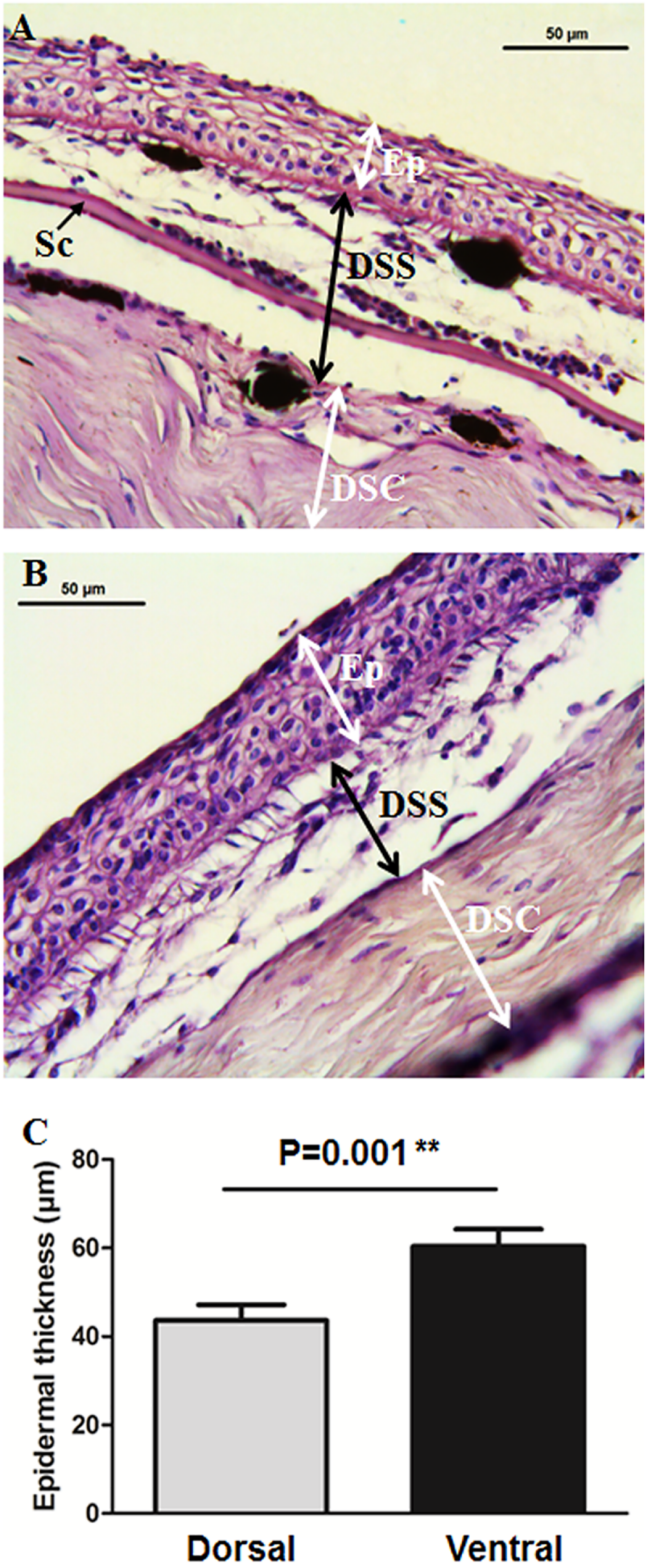
Epidermis thickness analysis. Representative images of dorsal (A) and ventral (B) skin from gilthead seabream stained with PAS. C. Differences in the epidermis layer thickness (μm) between dorsal (grey bar) and ventral (black bar) skin of gilthead seabream. Bars represent the mean ± SE (n = 6). P value resulting from Student t-test comparing both groups is indicated. DSC, dermis *stratum compactum*; DSS, dermis *stratum spongiosum*; Ep, epidermis; Sc, scale.

**Fig 3:**
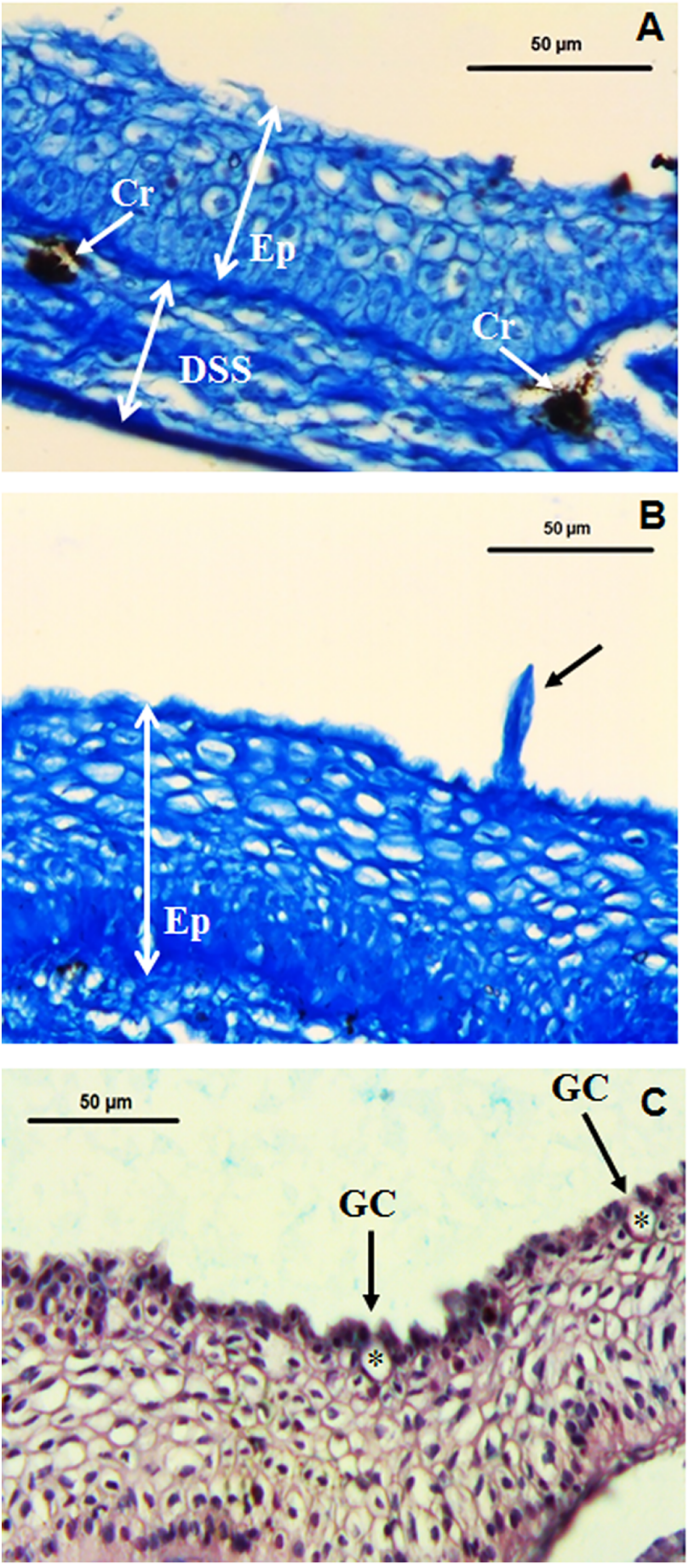
Specific structures in the fish skin. Representative images of dorsal (A) and ventral (B) skin from gilthead seabream stained with Mallory’s trichrome detailing a sensory cell (black arrow) and chromatophores (white arrows). Representative image of ventral skin (C) from gilthead seabream with goblet cells (asterisks) and skin mucus (in blue) stained with PAS. Cr, chromatophores; DSS, dermis *stratum spongiosum*; Ep, epidermis; GC, goblet cells.

Histological studies have become a powerful tool for tissue analysis since they were first used many years ago. Two articles suggested that epidermal thickness is mainly influenced by seasonal changes and/or sex [25,26], but neither could state so conclusively. In our results, light microscopic analysis showed that the epidermal layer is significantly less thick in the dorsal than in the ventral area (Figs 2 and 3). These differences in the skin of gilthead seabream were confirmed by image analysis (p = 0.001; Fig 2C). The staining pattern of epithelial cells, however, showed very little differences between both skin locations (Figs 2 and 3). Goblet cells (responsible for mucus production) are embedded in the epidermal layer but are predominant in the outer part of the epidermis (Fig 3C). In the epidermal layer, and externally, we identified a cell type with morphological characteristic of sensory cells round in shape and with an elongated protrusion (Fig 3B). Further studies will attempt to characterize their possible functions. Chromatophores appeared mainly in the upper layer of the dermis (*stratum spongiosum*) and in the ventral area (Figs 2A and 3A). Chromatophores are quite well studied to be mainly in the *stratum spongiosum* in both scaled and non-scaled skin [5]. Goblet cells have also been reported to exist in most teleosts [27], where they vary in number and/or appearance due to skin infections [28] as well varying between different parts of the body [25,29].

As regard epidermal thickness in the different regions of the fish body, there is no recent information, and only one review indicated that in benthic species the epidermal layer of ventral skin is often thicker [22]. Again, these differences in the epidermal thickness may be related with the ability of pathogens to colonize or cause skin lesions or ulcers in some regions of the skin rather in others.

### SEM study revealed different cell size and area of apical microridges of epidermis cells from dorsal and ventral skin

**Fig 4:**
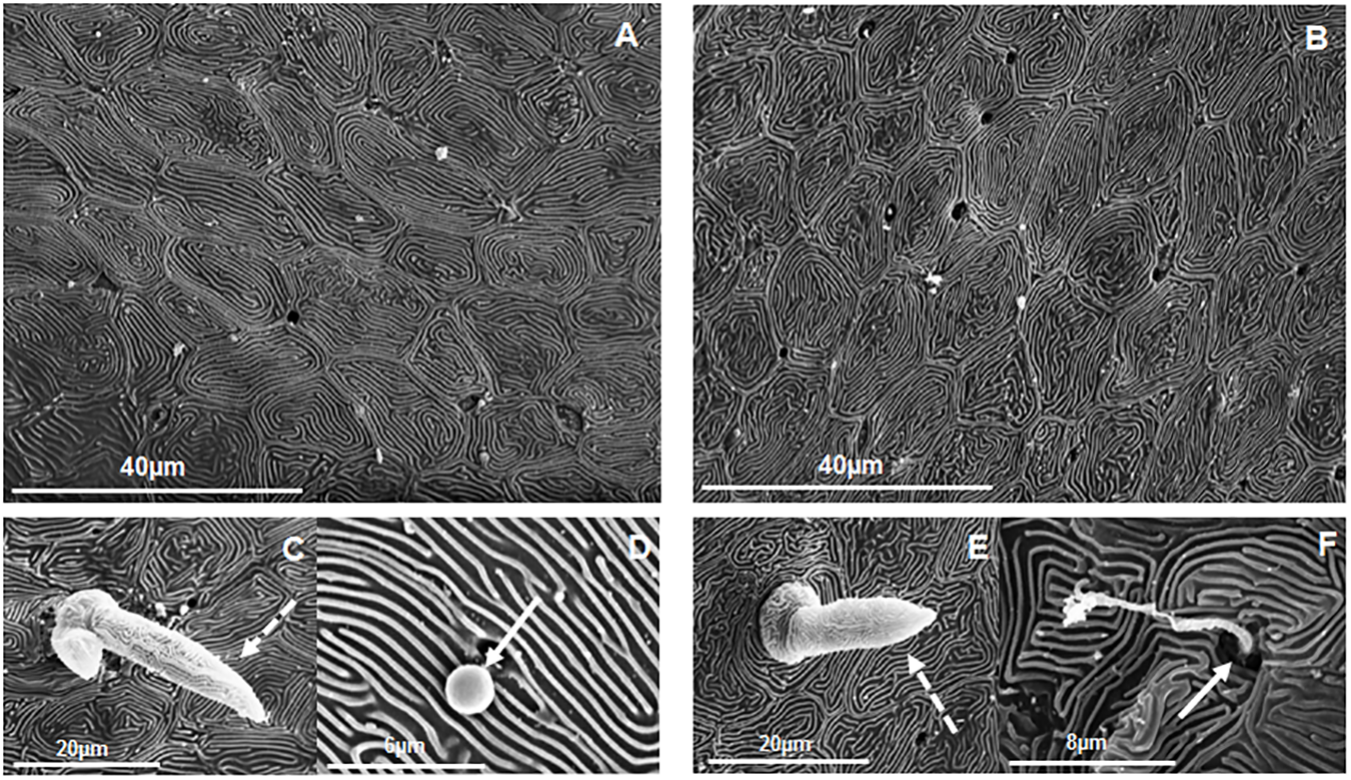
Analysis of fish skin by scanning electron microscopy (SEM). Representative images of dorsal (A, C, D) and ventral (B, E, F) skin from gilthead seabream studied by SEM. Sensory cells (discontinuous arrows) (C, E) and skin mucus secretions (continuous arrows) (D, F) are detailed.

**Fig 5:**
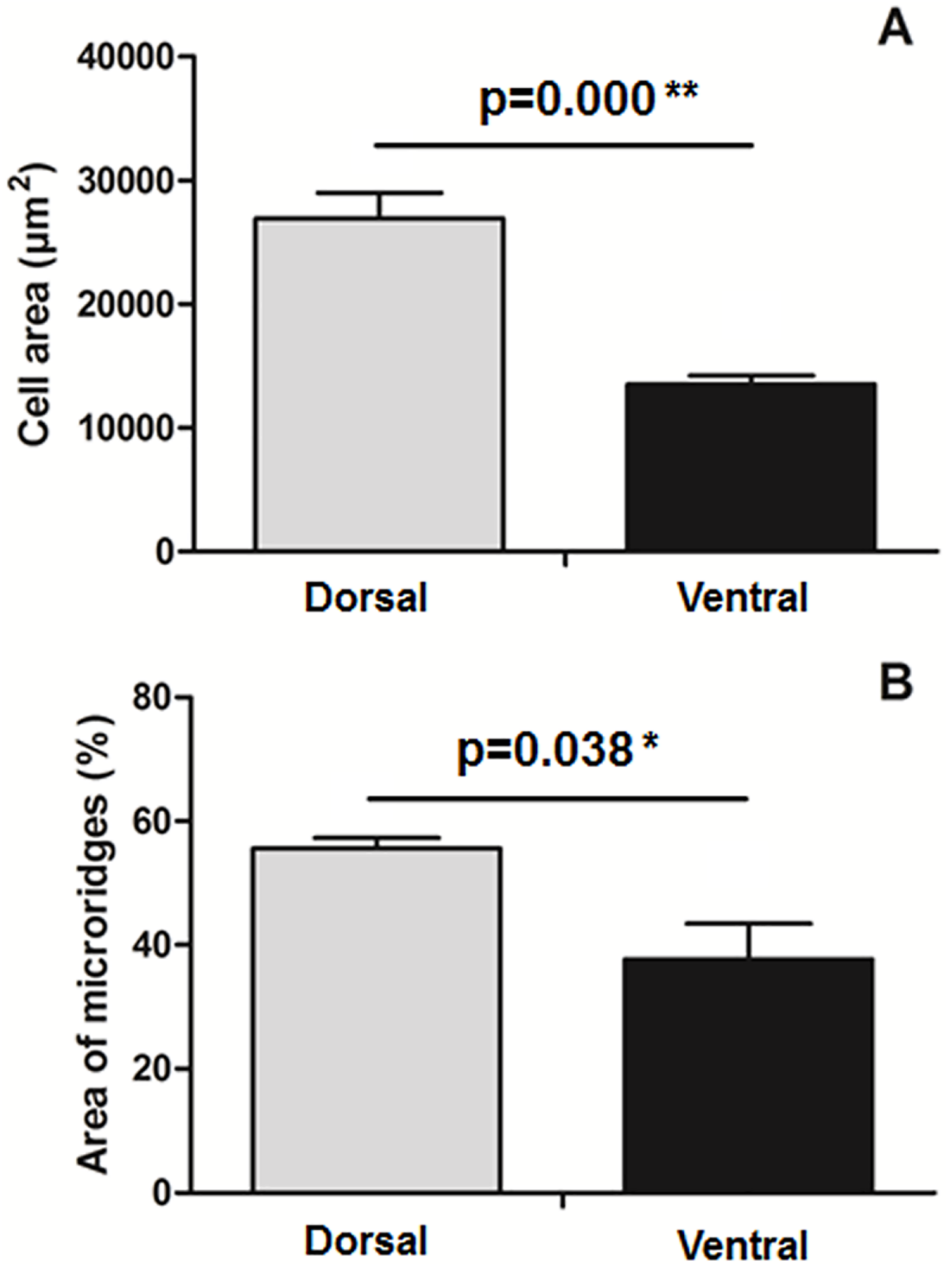
Analysis of cell area and area of microridges. Cell area (A) expressed in μm^2^, and area of microridges (B) expressed as percentage (%), in the dorsal (grey bars) and ventral (black bars) skin of gilthead seabream determined by analysis of SEM images. Bars represent the mean ± SE (n = 6). P value resulting from the Student t-test comparing both groups is indicated.

The dorsal and ventral skin of gilthead seabream was studied by SEM (Fig 4). Interestingly, the cell area (Fig 5A) and the area occupied by microridges (Fig 5B) are larger in the dorsal region than in the ventral region. There is no previous information about the differences in cell size or in the area of microridges for the apical part of the cells, except one work that describes preliminary data on microridges in wound healing [30]. It would be interesting to investigate alterations in the same since they seem to be involved not only in mechanical protection but also in the capacity to hold the skin mucus [4], and therefore in a better mucosal immune barrier.

### Gene expression in dorsal-ventral skin

**Fig 6:**
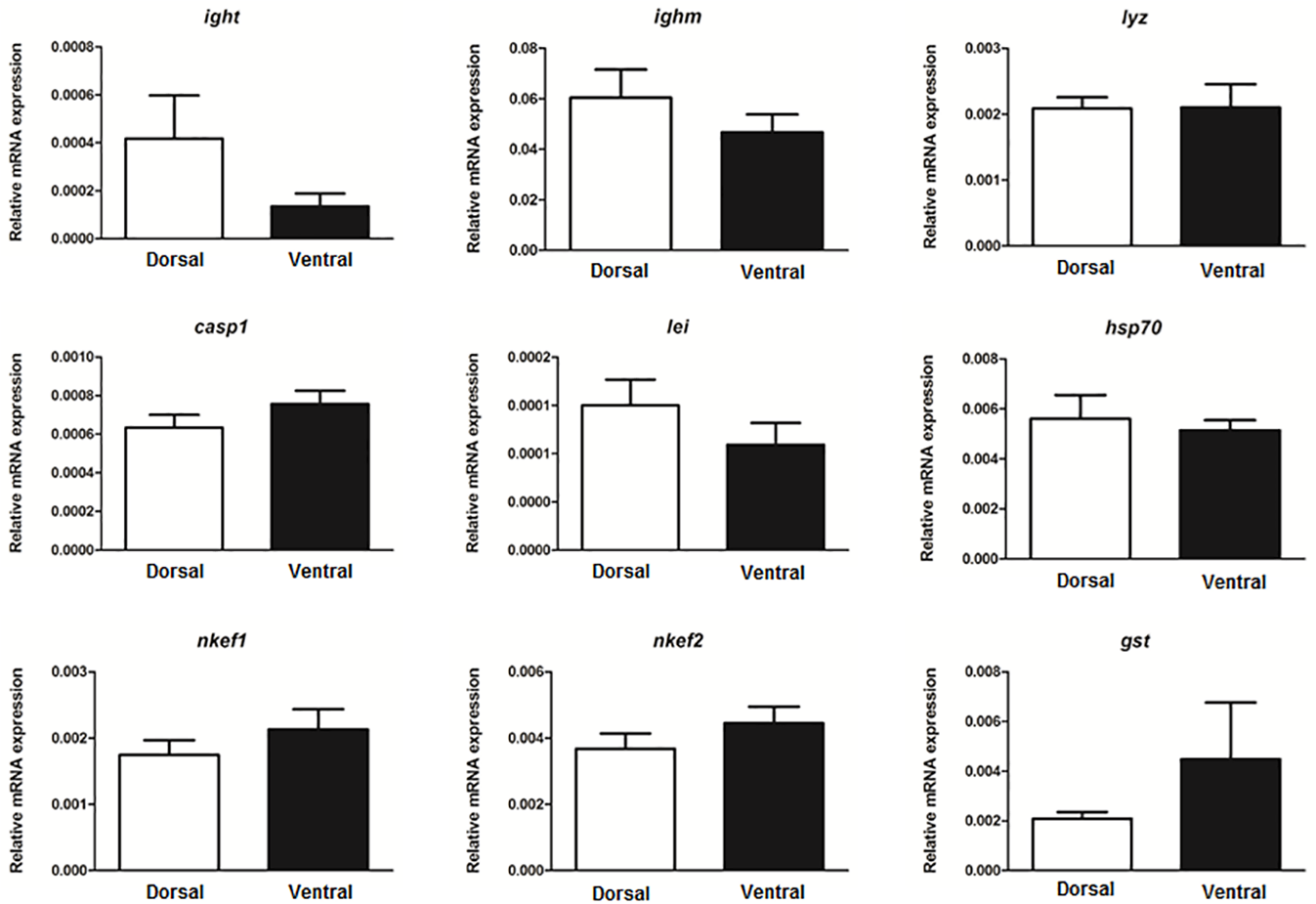
Gene expression study. The gene expression profile of several immune-markers determined in gilthead seabream skin. The constitutive mRNA level of each gene was studied by qPCR in the dorsal (white bars) and ventral (black bars) skin. Bars represent the mean ± SE (n = 4) of gene expression relative to the expression of the endogenous *ef1a* and *rps18* genes.

Previous studies have pointed to the different susceptibilities of dorsal and ventral skin after incubation with pathogens [18,19]. For this reason, we chose molecules previously reported in skin mucus of teleost, such as the immunoglobulins, *ighm* and *ight* [31], which are the major components of the adaptive immune system; as well as other important immune-related markers such as *lyz*, *casp1*, *lei*, *hsp70*, *nkef1*, *nkef2* and *gst*, which were found at proteomic level in our previous skin mucus studies [10,14]. The greatest differences between dorsal and ventral regions at transcript level were observed in the immunoglobulin specialized in mucosal immunology [32], *ight*, with lower levels in the ventral skin than in the dorsal region (Fig 6). A recent study reported the important up-regulation of *ight* in the skin of gilthead seabream after a diet of immunostimulants (mostly probiotics) [33], but, unfortunately, without differentiating between dorsal and ventral skin. Overall, our results show no differences in the expression of the selected genes in the dorsal and ventral skin of gilthead seabream (Fig 6). Although no differences in this respect were observed between ventral and dorsal skin in the present study, knowledge of constitutive transcript levels in both regions may be useful for saying the susceptibility of specific regions to stressors, as we showed for ventral skin in a previous study regarding the cytokine expression profile [19]. In addition, a previous study in European sea bass identified *lyz*, *casp1*, *lei*, *hsp70*, *nkef1*, *nkef2* and *gst* in skin mucus and evaluated their expression in the skin [10], so these molecules seem to be conserved across species.

### Dorsal and ventral skin regions have different healing rate

**Fig 7:**
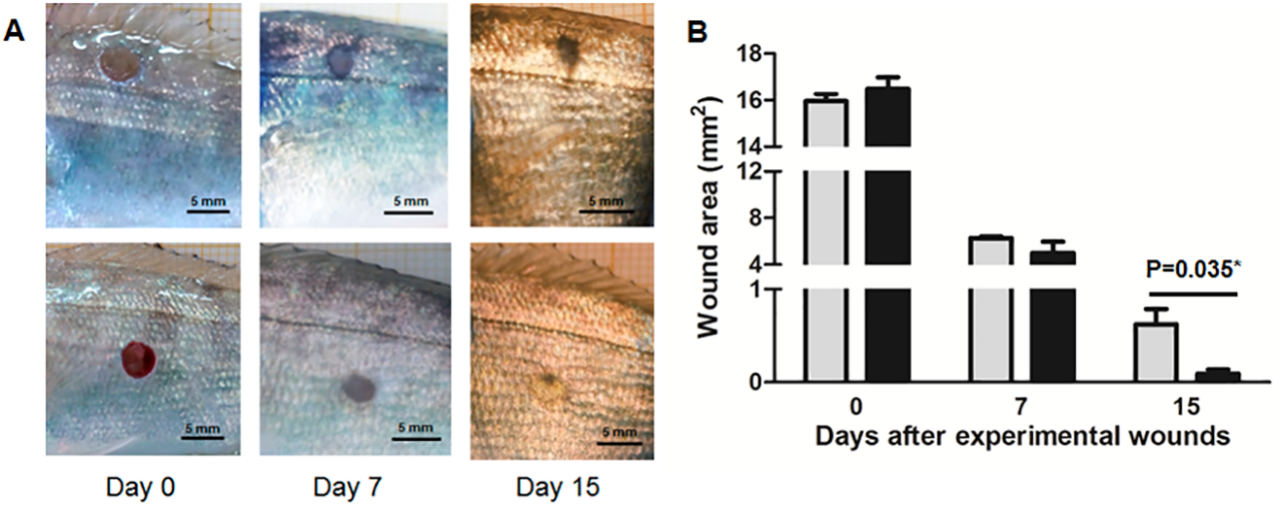
Experimental wounds and healing rate. Time-lapsed images (A) and area in mm^2^ (B) from experimental dorsal (grey bars) and ventral (black bars) skin wounds of gilthead seabream after 0, 7 and 15 days. Bars represent the mean ± SE (n = 3). P value resulting from the Student t-test comparing both groups is indicated.

On a previous study we demonstrated notable differences in the cytokine production between dorsal and ventral skin regions of gilthead seabream after pathogen and/or bacterial probiotic exposure [19]. In the present study, we have first demonstrated that both regions exhibit some physiological differences. Thus, our time-lapsed visual observations illustrated that the ventral skin region showed a faster rate of wound healing than the dorsal skin region of gilthead seabream (Fig 7A). Further, the image analysis of the wounds demonstrated significant differences in healing rate between both regions, with the wound totally closed after 15 days in the ventral skin region of gilthead seabream but still open in the dorsal region (Fig 7B). In spite of its importance due to the high amounts of microorganisms (both pathogenics and opportunistics) present normally in aquatic environments, the wound healing has been scarcely studied in fish [34]. Concretely, in gilthead seabream some studies have previously reported the scale regeneration under diet challenge by transcriptomic approach [35], the skin regeneration by a proteomic approach [36], and the role of collagen specific motifs in wound healing [37]. However, to the best of our knowledge, this is the first time that important differences between dorsal and ventral regions in wound healing are confirmed in a fish species.

## Conclusion

A ethicting gilthead seabream skin cells is presented and the cell cycle is analysed by flow cytometry in both dorsal and ventral regions of skin. Furthermore, analysis of epidermal 6 thickness, cell size and the area occupied by microridges revealed greater epidermal thickness but lower cell size and area of microridges in the ventral region compared to the dorsal region of skin of gilthead seabream. Furthermore, the transcriptomic analysis showed little change between both regions. Finally, we have demonstrated greater ratio of wound healing in the ventral region than in the dorsal region of the skin of gilthead seabream. These techniques represent useful tools for use in future studies of skin alterations and biology, while the study has also provided new insights into the skin structure and skin regeneration in teleost fish.

## Supporting information

S1 Fig Representative images of wounds in the dorsal (A) and ventral (B) skin of gilthead seabream.
(TIF)
