# Supplementary figures and images for: Dorso-ventral skin characterization of the farmed fish gilthead seabream (Sparus aurata)
Source: PLoS One. 2017 Jun 30;12(6):e0180438. doi: 10.1371/journal.pone.0180438 (PMC5493399; doi:10.1371/journal.pone.0180438)

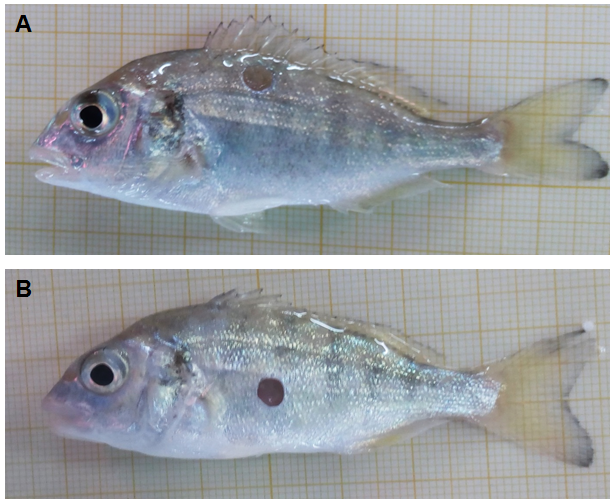

Supplement: S1 Fig — Representative images of wounds in the dorsal (A) and ventral (B) skin of gilthead seabream. (TIF) [file pone.0180438.s001.tif]
